# Real-world trends in rheumatic disease rates among pregnancies in Ontario, Canada: A repeated cross-sectional study

**DOI:** 10.1371/journal.pone.0332374

**Published:** 2025-09-15

**Authors:** Shenthuraan Tharmarajah, Zain Abideen, Swaleh Hussain, Sara J.T. Guilcher, Lisa M. McCarthy, Dharini Mahendira, Howard Berger, Mina Tadrous

**Affiliations:** 1 Leslie Dan Faculty of Pharmacy, University of Toronto, Toronto, Ontario, Canada; 2 ICES, Toronto, Ontario, Canada; 3 Institute for Better Health, Trillium Health Partners, Mississauga, Ontario, Canada; 4 Women’s College Hospital Research and Innovation Institute, Women’s College Hospital, Toronto, Ontario, Canada; 5 Division of Rheumatology, St. Michael’s Hospital, Toronto, Ontario, Canada; 6 Department of Medicine, University of Toronto, Toronto, Ontario, Canada; 7 Department of Obstetrics and Gynecology, Division of Maternal Fetal Medicine, St. Michael’s Hospital, Toronto, Ontario, Canada; 8 Department of Obstetrics and Gynecology, University of Toronto, Toronto, Ontario, Canada; Al Nasiriyah Teaching Hospital, IRAQ

## Abstract

**Objective:**

Despite increasing rheumatic disease prevalence among female individuals in Ontario, Canada, research is limited on whether this has translated to an increased proportion of pregnancies with rheumatic diseases. This study aimed to assess rheumatic disease rates among pregnant individuals in Ontario, Canada.

**Methods:**

Using healthcare administrative claims databases from ICES, the proportion of pregnancies with rheumatoid arthritis (RA), systemic lupus erythematosus (SLE), psoriatic arthritis (PsA), and ankylosing spondylitis (AS) from April 2011 to March 2021 were examined. The Mann-Kendall rank correlation test was used to test for trends in overall and individual rheumatic disease rates across the study period.

**Results:**

In total, 1,408,100 pregnancies were recorded from fiscal years 2011–2020 in Ontario. Of these, 4,392 involved patients with rheumatic diseases. The overall rheumatic disease rate among pregnant individuals increased by 24% from 28.3 to 35.2 pregnancies per 10,000 over the study period (p = 0.0157). RA rates increased 22% from 11.1 to 13.5 per 10,000 (p = 0.0056), while PsA rates increased 111% from 0.9 to 1.9 per 10,000 (p = 0.0157). SLE, AS, and multiple rheumatic disease rates did not increase significantly.

**Conclusion:**

The proportion of individuals with rheumatic diseases pursuing pregnancy in Ontario has increased, perhaps aided by advancements in disease diagnostics and treatment strategies. Further research is needed to link antirheumatic drug safety to pregnancy outcomes and explore other factors that may influence pregnancy rates among this patient population.

## Introduction

Rheumatic diseases, including rheumatoid arthritis (RA), systemic lupus erythematosus (SLE), psoriatic arthritis (PsA), and ankylosing spondylitis (AS), encompass a wide range of autoimmune disorders marked by chronic inflammation of the musculoskeletal system, connective tissue, and organs, with systemic effects driving disability and increasing risks of morbidity and mortality in affected patients [1–4]. As many of these diseases disproportionately affect female individuals of reproductive age, pregnancies in these individuals need to be closely monitored due to increased rates of pregnancy complications and adverse outcomes compared to the general population [5]. Further, rheumatic disease activity and pregnancy influence each other in various ways. RA typically improves during pregnancy but widely flares postpartum, whereas SLE, PsA, and AS either worsen or remain unchanged during pregnancy [6–11]. For these reasons, individuals of reproductive age living with rheumatic diseases may defer or avoid pregnancy due to concerns about antirheumatic medication safety, pregnancy complications and adverse outcomes, and potential transmission of rheumatic disease to their offspring [12].

While a growing body of research has explored pregnancy outcomes associated with rheumatic diseases, little is known about the number of individuals of reproductive age living with these conditions in Canada and whether this has shifted over time. Rheumatic disease prevalence has been rising among female individuals across all age groups in Ontario and Canada [13,14]. For instance, the age-standardized prevalence of RA among female individuals in Canada increased by 29%, from 382.8 per 100,000 in 1990 to 493.5 per 100,000 in 2019 [13]. Given this upward trend, it is important to better understand if the increasing prevalence of rheumatic diseases has also led to a higher proportion of affected pregnancies. This study aims to fill this knowledge gap by examining the prevalence of rheumatic diseases among pregnant individuals in Ontario, Canada’s most populous province.

## Methods

### Study design

We conducted a repeated cross-sectional study examining the annual rate of RA, SLE, PsA, and AS among individuals in Ontario, Canada who had been hospitalized for obstetrical delivery of a liveborn or stillborn singleton infant after 20 weeks of gestation, between April 1, 2011, and March 31, 2021.

### Data sources

We conducted this analysis using multiple linked population-based healthcare administrative claims databases housed at ICES in Toronto, Ontario. These included the Ontario Health Insurance Plan (OHIP) Claims History Database, the Canadian Institute for Health Information’s Discharge Abstract Database and National Ambulatory Care Reporting System, the OHIP Registered Persons Database, the Linked Delivering Mother and Newborns (MOMBABY) database, and the Better Outcomes Registry and Network (BORN) database. These datasets were securely linked using unique, encoded identifiers and analyzed at ICES, and they are routinely used to investigate drug safety and effectiveness due to their high reliability and validity [15]. The use of the data in this project is authorized under section 45 of Ontario’s Personal Health Information Protection Act (PHIPA) and does not require review by a Research Ethics Board.

### Study population

First, we identified in the BORN database all pregnant individuals who delivered a singleton infant (live or stillborn) between April 1, 2011, and March 31, 2021. We then identified pregnant individuals with RA, SLE, PsA, and/or AS in this population using validated algorithms involving combinations of healthcare utilization codes from hospitalization and physician diagnoses (S1 Table) [16]. As there are currently no validated algorithms for AS, the algorithm for RA was adapted similar to previous studies [16]. Only the first pregnancy after diagnosis for each unique individual was included in the overall cohort.

### Measures

To describe pregnancies according to the underlying rheumatic disease diagnosis, we stratified our study cohort into five subgroups: 1) RA subgroup, 2) SLE subgroup, 3) PsA subgroup, 4) AS subgroup, and 5) multiple diseases subgroup. Each of the first four study subgroups exclusively contained pregnant individuals with a recorded diagnosis for the respective disease, while those with multiple recorded diagnoses were included in the multiple diseases subgroup.

In our primary analysis, we reported the pregnancy-adjusted rates of rheumatic diseases (RA, SLE, PsA, and AS) overall among pregnant individuals in Ontario by fiscal year (beginning on April 1st and ending on March 31^st^ of each calendar year). We further stratified and reported the pregnancy-adjusted rates of each rheumatic condition among these individuals by fiscal year. Rates were reported per 10,000 total pregnancies, defined as the Ontario population of pregnant individuals who delivered a liveborn or stillborn singleton infant during the study period.

### Statistical analysis

Trends across years were evaluated separately for continuous and categorical variables. For continuous variables, general linear regression models were used with the variable of interest as the dependent variable and calendar year (treated as a continuous variable) as the independent variable to test for linear trends over time. Model assumptions, including normality and homoscedasticity of residuals, were checked using standard diagnostic plots.

For categorical variables, trend analysis depended on the nature of the variable. For binary categorical variables, the Cochran-Armitage trend test was applied to detect linear trends in proportions across ordered years. For ordinal categorical variables with more than two categories, the Jonckheere-Terpstra test was used to assess ordered differences across years without assuming linearity.

All statistical analyses were performed using SAS version 9.4 (SAS Institute Inc). Statistical significance was set at p < .05, and all tests were 2-tailed. Cells with <6 pregnant individuals were suppressed due to ICES privacy policies.

## Results

**Fig 1:**
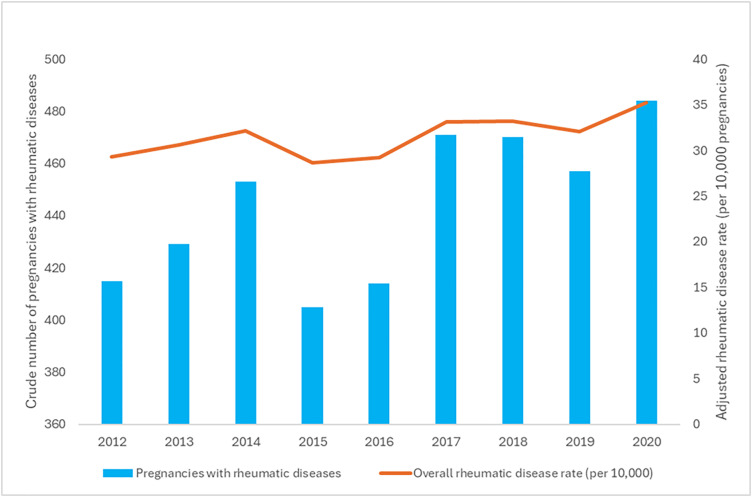
Crude number of pregnancies with rheumatic diseases and adjusted rheumatic disease rates per 10,000 pregnancies in Ontario, Canada from 2011 to 2020.

**Fig 2:**
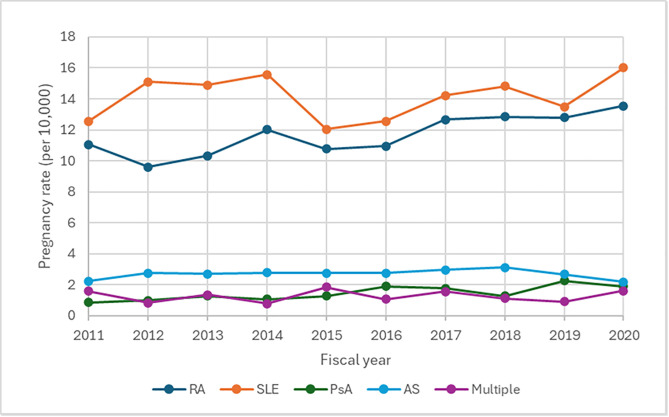
Stratified rheumatic disease rates per 10,000 pregnancies in Ontario, Canada from 2011 to 2020.

A total of 1,408,100 pregnancies were documented from fiscal years 2011–2020 in Ontario, Canada. Of these, 4,392 (0.3%) involved patients diagnosed with rheumatic diseases. The overall proportion of pregnancies involving individuals with rheumatic diseases significantly increased 24% from 28.3 pregnancies per 10,000 total pregnancies in fiscal year 2011 to 35.2 pregnancies per 10,000 total pregnancies in fiscal year 2020 (Fig 1) (p = 0.0157). The proportion of pregnancies involving the RA subgroup increased 22% from 11.1 per 10,000 in fiscal year 2011 to 13.5 per 10,000 in fiscal year 2020 (Fig 2) (p = 0.0056). The proportion of pregnancies involving the PsA subgroup also increased 111% from 0.9 per 10,000 in fiscal year 2011 to 1.9 per 10,000 in fiscal year 2020 (p = 0.0157). On the other hand, while the proportion of pregnancies involving the SLE subgroup increased 27% from 12.6 per 10,000 in fiscal year 2011 to 16.0 per 10,000 in fiscal year 2020, this increase was not statistically significant. Lastly, the proportion of pregnancies involving the AS and multiple rheumatic diseases subgroups remained stable across the study period at 2.2 per 10,000 and 1.6 per 10,000, respectively.

## Discussion

In this repeated cross-sectional study over a 10-year period from fiscal years 2011–2020, we examined the changing longitudinal patterns in rheumatic disease rates among pregnant individuals in Ontario, Canada. Our analysis found that the proportion of pregnancies involving individuals with rheumatic diseases has grown significantly over the study period. This was particularly driven by increases in the proportion of pregnancies involving individuals with RA and PsA. On the other hand, the proportion of pregnancies involving individuals with SLE, AS, and multiple rheumatic diseases has not changed significantly. Nonetheless, these statistics highlight the need for more research on rheumatic disease management during pregnancy and prenatal antirheumatic drug safety.

Over recent decades, rheumatic disease rates have risen in the general population in Ontario [17–19] and across Canada [14,20]. This is possibly due to population-related factors (e.g., population growth, aging, increasing life expectancy) as well as advances in healthcare (e.g., better diagnosis and case identification, improved survival, more effective disease management strategies) [19]. Therefore, our finding of an observed increase in the proportion of pregnancies involving with rheumatic diseases is in line with this trend, likely due to both true elevation in disease burden as well as ongoing development of updated clinical guidelines for managing rheumatic disease during pregnancy [21]. In terms of pregnancy-safe pharmacotherapy, safety data for the newer biological antirheumatic therapies, particularly tumor necrosis factor inhibitors, and their effects on pregnancy outcomes continues to accumulate, thus enabling their use during pregnancy. Finally, more comprehensive preconception counselling has allowed patients to plan and manage their pregnancies to optimize maternal and neonatal outcomes [21].

RA is unique from other rheumatic diseases as its disease activity can improve during pregnancy [6]. In 2019, a meta-analysis by Jethwa et al. using validated tools to assess disease severity such as the DAS28 found that of 204 RA pregnancies across 10 studies, disease activity improved in 123 pregnancies (60.3%), with an improvement range of 40.4% to 90%, although nearly half of all patients experienced a postpartum flare (46.7%). While patients with active RA face higher risks of adverse obstetric outcomes, those with well-controlled disease activity have also shown pregnancy outcomes comparable to the general population, including no increased fetal morbidity or fetal losses [22]. Better disease control may lead more individuals with RA to pursue pregnancy, possibly contributing in tandem with rising RA rates [18] to the rising proportion of pregnancies involving RA across our study period.

For SLE patients, pregnancy is generally unproblematic unless cytotoxic agents are used for disease management, but for those with a more severe disease profile, potential complications such as concomitant nephritis may arise during pregnancy [7]. In 2016, a prospective study by Buyon et al. found that among 385 American and Canadian patients with SLE, 81% had uncomplicated pregnancies, 5% of pregnancies ended in fetal or neonatal death, and severe maternal flares occurred in less than 3% of pregnancies. These findings suggest that pregnancy is relatively safe for those with inactive or stable SLE. However, the lack of any significant increase in the proportion of pregnancies involving SLE in our study suggests that perhaps decisions regarding conception are not necessarily based on maternal disease control and therapeutic management alone. Further studies are needed to explore the complex factors underlying decisions to pursue pregnancy in this patient population as well as assess the impact of rheumatic disease activity in those who choose to do so.

Considering PsA and AS, the introduction of biological antirheumatic therapies has significantly improved disease management and quality of life, and such advancements have in turn improved pregnancy outcomes for patients with these conditions [8]. In a 2017 study by Polachek et al. of 42 pregnancies in Toronto, 40 (95%) resulted in normal live births, and PsA disease activity was stable or remitted in 24 (58.5%) of cases during pregnancy. Furthermore, 21 (52.5%) of patients displayed this stability or improvement in disease activity postpartum as well, while 16 (40%) experienced worsening or stable high disease activity. These findings suggest that pregnancy outcomes for those with well-controlled PsA activity are favorable, possibly contributing alongside rising PsA rates [19] to the observed increase in the proportion of pregnancies involving PsA in our study.

On the other hand, the proportion of pregnancies involving AS remained stable across the study period. It is noteworthy that AS primarily affects the spine, whereas other rheumatic diseases affect multiple joints or organs. This localized manifestation of AS, in tandem with emerging novel biologic therapies, enables targeted treatment options that significantly reduce inflammation and enhance patient quality of life as well as pregnancy outcomes. However, our findings suggest that the proportion of pregnancies involving AS has not kept up with the more rapid increase in prevalence among female individuals of reproductive age in Ontario [17]. This suggests that as with the SLE subgroup, more research is needed on the decision-making process regarding pregnancy in this patient population.

To explore patient and provider perceptions of rheumatic disease management during pregnancy, Clowse et al. engaged with 36 rheumatology clinicians and 15 individuals with SLE at various stages of pregnancy in the US [23]. Patients reported feeling intimidated by their rheumatologists and were hesitant to engage in guilt-and fear-laden conversations about pregnancy and its associated risks, preferring instead to avoid confrontation. Notably, all participants in the study researched each new prescription online before starting it due to a distrust of clinicians. Participants also admitted to not being upfront with their rheumatologist about their strong desire for pregnancy, which superseded any concerns about the risks involved. On the other hand, Mills et al. found differing views in their investigation on perceptions of pregnancy and lactation among those with autoimmune or inflammatory diseases [12]. In a single-center retrospective cohort study of 154 subjects, the primary concerns were the potential impact of perinatal medication use on both fetal and maternal health, along with the ability to care for their offspring post-pregnancy, potentially due to disease activity worsening during lactation. More than 65% of individuals expressed concern about the adverse impact of medication use during pregnancy on the fetus. Additionally, 52% of patients stated that their diagnosis had negatively affected their views on pregnancy, with 30% deciding altogether not to pursue pregnancy after their diagnosis. Taken together, these studies suggest that individuals with rheumatic diseases fall on a spectrum of varying perceptions of pregnancy as well as its associated risks and outcomes. This variability underscores the need for more comprehensive research to explore patient and provider perspectives.

A strength of this study is its use of population-based healthcare administrative claims data to identify a cohort of pregnant individuals with rheumatic diseases. While this approach allows for a robust population-level analysis, a potential limitation is that not all individuals with RA, SLE, and PsA may have been captured despite employing validated coding algorithms from previous research. Additionally, there are currently no validated algorithms for identifying AS, leading us to adapt the RA algorithm, as done in previous studies [16]. However, this adaptation may have missed some cases of AS due to potential misclassification by less experienced clinicians or missing documentation from specialists. Furthermore, our analysis focused solely on the four most prevalent rheumatic conditions, excluding rarer conditions such as juvenile idiopathic arthritis, reactive arthritis, scleroderma, and Sjögren’s disease, potentially underestimating the overall burden of rheumatic diseases in pregnancy in Ontario. Finally, as our study is limited to data from Ontario, its results may not be generalizable to describe rheumatic disease rates among pregnancies across other regions of Canada. Further research using data from other provinces is needed to assess whether similar trends are observed nationwide.

Findings from this research can inform future studies on pregnancy patterns among individuals affected by rheumatic diseases. Future studies might consider pregnancy rates specifically among populations of reproductive age with rheumatic diseases and investigate how these rates have changed with time, considering factors such as disease severity, emerging therapeutic advancements, and proactive preconception planning by patients and their physicians. Given that the prevalence of rheumatic diseases among female individuals in Ontario continues to increase, additional research examining the impact of disease severity and antirheumatic drug safety on pregnancy outcomes can greatly benefit this patient population in achieving safe pregnancies. From a clinical perspective, such findings could further bolster and update current prescribing guidelines as well as aid and inform clinicians in making critical decisions in tandem with their patients.

**Significance and Innovations**

- The proportion of individuals with rheumatic diseases pursuing pregnancy in Ontario has significantly increased.
- The proportions of pregnant individuals with rheumatoid arthritis and psoriatic arthritis have especially increased.
- More research is needed to demonstrate antirheumatic drug safety in pregnancy and implications for pregnancy rates among this population.

## Supporting information

S1 Table ICD code definitions for rheumatic conditions. (DOCX)
