# Supplementary material for: Real-world trends in rheumatic disease rates among pregnancies in Ontario, Canada: A repeated cross-sectional study
Source: PLoS One. 2025 Sep 15;20(9):e0332374. doi: 10.1371/journal.pone.0332374 (PMC12435662; doi:10.1371/journal.pone.0332374)
Supplement: S1 Table — (DOCX) [file pone.0332374.s001.docx]

| **Rheumatic condition** | **Definition** |
| --- | --- |
| Rheumatoid arthritis (RA) | ≥3 outpatient visits with OHIP rheumatoid arthritis diagnosis codes (714) within 2 years, with ≥1 by a rheumatologist/internal medicine specialist/orthopedic surgeon  OR  ≥1 hospital discharge abstract with rheumatoid arthritis listed as reason for hospitalization or co-morbid condition (primary, secondary, and type 3 diagnosis codes)  ICD-9: 714  ICD-10: M05, M06 |
| Systemic lupus erythematosus (SLE) | ≥2 outpatient visits at least 2 months apart over 2 years with OHIP systemic lupus erythematosus diagnosis codes (710.0) by any physician  OR  >1 outpatient visit with OHIP systemic lupus erythematosus diagnosis codes (710.0) by a rheumatologist/internal medicine specialist  OR  ≥1 hospital discharge abstract with systemic lupus erythematosus listed as reason for hospitalization or co-morbid condition (primary, secondary and type 3 diagnosis codes)  ICD-9: 710.0  ICD-10: M32 |
| Ankylosing spondylitis (AS) | ≥3 outpatient visits within 3 years with OHIP ankylosing spondylitis diagnosis codes (720), ≥1 by a rheumatologist/internal medicine specialist  OR  ≥1 hospital discharge abstract with ankylosing spondylitis listed as reason for hospitalization or co-morbid condition (primary, secondary, and type 3 diagnosis codes)  ICD-9: 720.0  ICD-10: M45 |
| Psoriatic arthritis (PsA) | ≥1 hospitalization (DAD) with a primary or secondary discharge diagnosis (DXCODE1 or DXCODE2) of psoriatic arthritis  OR  ≥1 outpatient visits (OHIP) with a diagnosis of psoriasis (includes visits to all types of PC and/or specialist physicians) AND ≥2 primary care visits (OHIP) with a diagnosis of spondyloarthritis, with ≥1 of these diagnoses by a rheumatologist or internist ever  ICD-9: 696.0  ICD-10: L40.5, M07.0, M07.1, M07.2, M07.3, M09.0 |
